# Systemic IgG4-related disease with extensive peripheral nerve involvement that progressed from localized IgG4-related lymphadenopathy: an autopsy case

**DOI:** 10.1186/1746-1596-9-41

**Published:** 2014-02-21

**Authors:** Masayoshi Fujii, Yasuharu Sato, Nobuya Ohara, Kenji Hashimoto, Haruhiko Kobashi, Yoshinobu Koyama, Tadashi Yoshino

**Affiliations:** 1 Department of Pathology, Okayama University Graduate School of Medicine, Dentistry and Pharmaceutical Sciences, 2-5-1 Shikata-cho, Kita-ku, Okayama 700-8558, Japan; 2 Department of Diagnostic Pathology, Japanese Red Cross Okayama Hospital, 2-1-1 Aoe, Kita-ku, Okayama 700-8607, Japan; 3 Department of Gastroenterology, Japanese Red Cross Okayama Hospital, 2-1-1 Aoe, Kita-ku, Okayama 700-8607, Japan; 4 Department of Collagen and Rheumatic diseases, Japanese Red Cross Okayama Hospital, 2-1-1 Aoe, Kita-ku, Okayama 700-8607, Japan

**Keywords:** IgG4, IgG4-related disease, IgG4-related lymphadenopathy, IgG4-related perineural disease, Systemic progression, Peripheral nerve involvement

## Abstract

A 77-year-old man, with a lengthy medical history of chronic dysuria, constipation, hypertension, myocardial infarction, and a submandibular lymphadenopathy that was excised 3 years ago, was hospitalized due to elevated liver enzyme levels. He demonstrated hypergammaglobulinemia, hyperproteinemia, high levels of IgG and IgG4, eosinophilia, sclerosing cholangitis, and retroperitoneal fibrosis. He was diagnosed with IgG4-related disease (IgG4-RD). While hospitalized, he had several episodes of syncope while standing and was diagnosed with autonomic nerve dysfunction. Thirty days after hospitalization, he died of nonocclusive mesenteric ischemia (NOMI). Post-mortem, his submandibular lymphadenopathy lesion was diagnosed with progressively transformed germinal center (PTGC)-type IgG4-related lymphadenopathy. At autopsy, small and large intestines showed mucosal necrosis and the wall muscles of the transverse to sigmoid colon were necrotic. The sigmoid colon was fibrotic and infiltrated with numerous IgG4^+^ plasma cells and eosinophils; infiltration into Auerbach’s plexus was also observed. The IgG4-RD lesions were also detected in the mesentery of the sigmoid colon, retroperitoneal soft tissue, abdominal aorta, liver, extrahepatic bile duct, bilateral lungs, bilateral kidneys, urinary bladder, prostate, epicardium, bilateral coronary arteries, and lymph nodes. Interestingly, infiltration into the lesions was most notable around the peripheral nerves in every organ. Thus, this case describes an IgG4-RD that progressed from PTGC-type IgG4-related lymphadenopathy to systemic IgG4-RD, suggesting that IgG4-RD may affect many organs through peripheral nerve involvement.

**Virtual slide:**

The virtual slides for this article can be found here: http://www.diagnosticpathology.diagnomx.eu/vs/9995992971155224.

## Introduction

Immunoglobulin G4-related disease (IgG4-RD) comprises a recently recognized systemic syndrome that is characterized by mass-forming lesions, mainly in exocrine tissues, which consist of lymphoplasmacytic infiltrations and sclerosing fibrosis [1,2]. IgG4-RD frequently involves the lacrimal gland, salivary gland, lymph node, lung, pleura, pancreas, bile duct, liver, kidney, aorta, retroperitoneum, and skin [1]. Numerous IgG4^+^ plasma cells are typically found in the affected tissues, and the patient’s serum IgG4 level is increased. Generally, if the histological IgG4^+^/IgG^+^ cell ratio is greater than 40%, the diagnostic possibility of IgG4-RD is high, but there are also many “non-IgG4-related” diseases such as multicentric Castleman’s disease, rheumatoid arthritis, or other autoimmune diseases that need to be included in the differential diagnosis [1,3,4].

The lymph node lesions of IgG4-RD can be subdivided into at least 5 histological subtypes, including the multicentric Castleman’s disease-like, reactive follicular hyperplasia-type, interfollicular expansion and immunoblastosis, progressively transformed germinal center (PTGC)-type, and inflammatory pseudotumor (IPT)-like [1,4]. The PTGC-type usually presents with uniform clinicopathological features of asymptomatic, localized, submandibular lymphadenopathy that persists and/or relapses, and sometimes progresses to extranodal lesions or systemic disease [4,5]. Recently, a few papers have described peripheral nerve involvement in IgG4-RD, especially around the trigeminal nerve and optic nerve branches [6,7].

We present a rare autopsy case of IgG4-RD that began as PTGC-type IgG4-related lymphadenopathy and progressed into a systemic disease by infiltration around the peripheral nerves of many organs.

## Case presentation

A 77-year-old man, with a lengthy history of chronic dysuria, constipation, hypertension, and a previous myocardial infarction that was treated with a coronary stent, developed a right submandibular mass 3 years prior to his most recent admission and underwent an excisional biopsy. As a result, he was diagnosed with reactive follicular hyperplasia with PTGC. During the intervening period, the patient had slight fatigue, loss of appetite, and lightheadedness. At a regular check-up, serological tests showed high levels of liver enzymes and he was admitted to a local hospital. The serological examination results revealed that serum IgG, IgG4, and IgE levels were notably elevated; we also noted eosinophilia, hyperproteinemia, hypoalbuminemia, low albumin/globulin ratio, hyponatremia, and elevations of liver enzymes (Table 1).

**Table 1:** Serological examination results

		**Normal range**			**Normal range**
White blood cell count	11,300/μL	(4,700–7,900)	BUN	14.0 mg/dL	(8.0–18.0)
Neutrophils	37.6%	(25–45)	Cr	0.9 mg/dL	(0.6–1.1)
Lymphocytes	19.0%	(4.0–7.0)	Na	122 mEq/L	(138.0–146.0)
Monocytes	4.3%	(48–61)	K	4.0 mEq/L	(3.8–5.1)
Eosinophils	38.4%	(1.0–5.0)	Cl	90 mEq/L	(98.0–108.0)
Basophils	0.7%	(0.0–1.0)	Ca	7.8 mg/dL	(8.4–10.2)
Red blood cell count	403 × 10^4^/μL	(411–539)	P	3.3 mg/dL	(2.5–4.5)
Hemoglobin	12.9 g/dL	(14.0–15.8)	AST	88 U/L	(8.0–40.0)
Hematocrit	37%	(40.2–52.4)	ALT	160 U/L	(5.0–35.0)
Platelet count	19.0 × 10^4^/μL	(13.0–40.0)	LDH	164 U/L	(125–250)
Total protein	9.4 g/dL	(6.5–8.0)	ALP	915 U/L	(110–360)
Albumin	2.3 g/dL	(3.8–5.3)	LAP	100 U/L	(16.0–70.0)
A/G ratio	0.32	(1.42–2.55)	γGTP	67 U/L	(0.0–15.0)
IgA	294 mg/dL	(113–463)	ChE	162 U/L	(168–470)
IgG	4,813 mg/dL	(837–1,825)	T-bil	0.2 mg/dL	(0.2–0.8)
IgG4	1,750 mg/dL	(4–108)	D-bil	0.1 mg/dL	(0.0–0.2)
IgM	28 mg/dL	(57–288)	T-chol	117 mg/dL	(168–470)
IgE	13,136 IU/mL	(0.0–170)	CRP	<0.3 mg/dL	(0.0–0.3)

*Abbreviations*: *A/G ratio* albumin/globulin ratio, *Ig* immunoglobulin, *BUN* blood urea nitrogen, *Cr* creatinine, *Na* sodium, *K* potassium, *Cl* chlorine, *Ca* calcium, *P* phosphorus, *AST* aspartate aminotransferase, *ALT* alanine aminotransferase, *LDH* lactate dehydrogenase, *ALP* alkaline phosphatase, *LAP* leucine aminopeptidase, *γGTP* γ-glutamyl transpeptidase, *ChE* cholinesterase, *T-bil* total bilirubin, *D-bil* direct bilirubin, *T-chol* total cholesterol, *CRP* C-reactive protein.

As part of the differential diagnosis of high gammaglobulinemia, M-proteins were not detected in the globulin fraction and the immunoglobulin exhibited a polyclonal pattern, excluding hematopoietic diseases such as multiple myeloma. The hyponatremia was considered to be a syndrome of inappropriate antidiuretic hormone secretion, considering the patient’s low osmotic blood pressure, his high urinary osmotic pressure, and the lack of serum renin elevation. After hospitalization, the patient’s dysuria and constipation were exacerbated. After 2 weeks of hospitalization, he experienced 3 episodes of syncope while standing up. The cause of the syncope was determined to be orthostatic hypotension, and no hormonal abnormalities were detected in his catecholamine, thyroid hormone, and renin-aldosterone-angiotensin levels. Using cardiac metaiodobenzylguanidine scintigraphy, he was diagnosed with severe autonomic nerve dysfunction.

**Figure 1:**
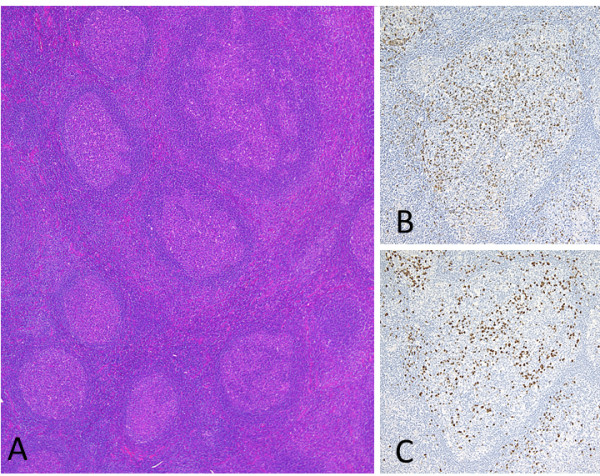
**Submandibular lymph node. A**: Several progressively transformed germinal centers (PTGC) are observed at a low magnification. **B-C**: Immunostaining for IgG **(B)** and IgG4 **(C)**. Many plasma cell infiltrates are seen, and the IgG4^+^/IgG^+^ cell ratio is more than 40%, which is consistent with PTGC-type IgG4-related lymphadenopathy.

While the patient was hospitalized, the submandibular lymph node specimen that was excised 3 years previously was reviewed and numerous IgG4^+^ plasma cells were seen, immunohistologically, within the germinal centers. There was no evidence of kappa or lambda immunoglobulin light chain restriction by *in situ* hybridization. This finding led us to re-diagnose his previous lymphadenopathy as PTGC-type IgG4-related lymphadenopathy (Figure 1). Thus, combined with his present findings, including hypergammaglobulinemia, high serum levels of IgG and IgG4, and eosinophilia, he was diagnosed with IgG4-RD.

Cranial computed tomography and magnetic resonance imaging (MRI) indicated inflammation of the pituitary gland, and a magnetic resonance cholangiopancreatography revealed mild narrowing of the extrahepatic and intrahepatic bile ducts in the portal area. This was considered as evidence of sclerosing cholangitis. Abdominal MRI also showed a retroperitoneal mass that was 2 cm in diameter. These lesions were also considered as indications of IgG4-RD.

Following this diagnosis, the patient was scheduled to begin steroid therapy. However, prior to starting the therapy, 30 days after hospitalization, the patient had abdominal distention, nausea, and vague abdominal tenderness that developed into a sharp abdominal pain. He also exhibited low blood pressure, tachycardia, and marked metabolic acidosis, eventually going into cardiac arrest. The patient was resuscitated and, upon suspicion of ileus or non-occlusive intestinal ischemia, he underwent emergent abdominal surgery. The patient was subsequently determined to have inoperable, massive necrosis of the small and large intestines. Some hours later, he succumbed to shock and an exacerbation of metabolic acidosis.

**Figure 2:**
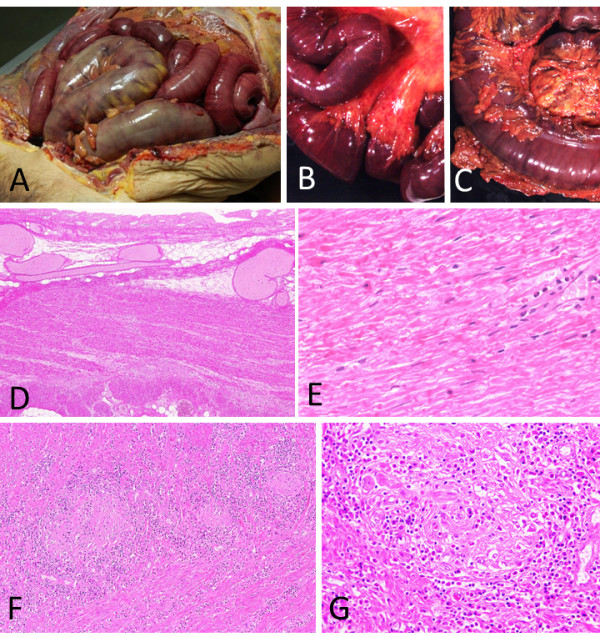
**Intestines. A-C**: Macroscopic features of the intestines (**A**: peritoneal overview, **B**: small intestine, **B**: large intestine). The small and large intestines are diffusely congested. **D-G**: Microscopic features of the sigmoid colon (**D**, **F**: low magnification; **E**, **G**: high magnification). The mucosa is necrotic **(D)**, and diffuse coagulative necrosis of the proper muscle layer is seen **(E)**. The proper muscle layer is infiltrated with inflammatory cells **(F)**, and the intramuscular Auerbach’s nerve plexus is destroyed **(G)**.

An autopsy was performed 8 h after death. A central nervous system examination was not permitted by the family; thus, the autopsy was restricted to the chest, abdomen, and spinal cord. The peritoneal examination revealed the presence of approximately 550 mL of sanguineous ascites. The small and large intestines were diffusely dark red to black, but no mechanical obstruction was observed; the intestinal tract was filled with bloody stool. Histologically, the intestinal mucosa was diffusely necrotic, and the muscle layers were also necrotic. The necrosis of the colonic muscle layers was coagulative, focal, and patchy along the transverse and descending colon, whereas there was diffuse necrosis in the sigmoid colon. In addition to necrosis, the sigmoid colon wall was infiltrated by numerous plasma cells and eosinophils. These lesions were primarily located in the smooth muscle of the colon wall and in the intramuscular nerve plexus of Auerbach; plasma cell infiltration, through the epineurium, into the plexus was also observed (Figure 2). Similar infiltration of plasma cells and eosinophils into the duodenal wall and nerve plexus was also observed.

**Figure 3:**
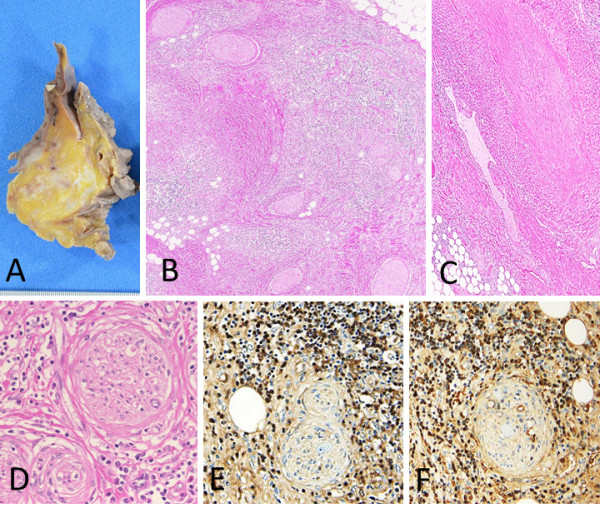
**Mesentery of the sigmoid colon. A**: Macroscopically, patchy fibrotic lesions are seen between fat tissues. **B**: Fibrosis and dense inflammatory cells are patchily distributed. The inflammatory cells are distributed preferentially around the peripheral nerves (arrowhead). **C**: Obliterative phlebitis and organization of the accompanying artery are seen. **D-F**: Immunostaining for IgG **(E)** and IgG4 **(F)** in the peripheral nerves. The IgG4^+^/IgG^+^ cell ratio is more than 40%.

Macroscopic obstruction of the major arterial branches of the abdominal aorta, including the celiac artery, and the superior and inferior mesenteric arteries was not apparent. The mesentery of the sigmoid colon was diffusely thickened, with patchy fibrosis that occasionally showed a storiform pattern, and infiltration of inflammatory cells, including numerous plasma cells, eosinophils, and lymphocytes. The plasma cells were preferentially distributed around the peripheral nerves, and several plasma cells had infiltrated the nerve fascicles. Most of these plasma cells were positive for IgG4, with an IgG4^+^/IgG^+^ cell ratio of more than 40%, histologically consistent with IgG4-RD. Within this lesion, some of the small veins in the sigmoid colon mesentery showed obliterative phlebitis and a few of the accompanying arteries were organized (Figure 3).

**Figure 4:**
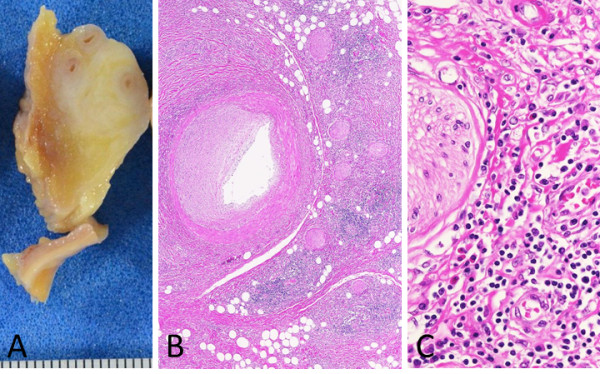
**A retroperitoneal mass around the left internal iliac artery. A**: Fibrotic nodules approximately 2 cm in diameter are seen. **B**: Fibrosis and dense inflammatory cells are patchily distributed around the peripheral nerves, and obliterative phlebitis is seen. **C**: At high magnification, infiltrates of plasma cells and eosinophils are seen.

**Figure 5:**
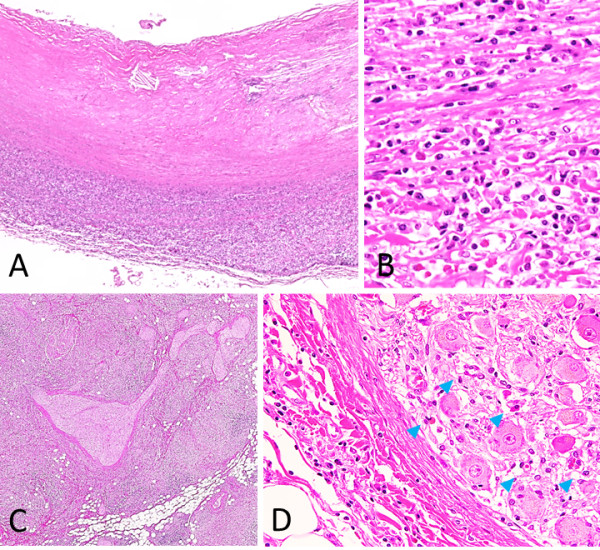
**Abdominal aorta (A, B) and retroperitoneum (C, D). A**: At low magnification, inflammatory cell infiltrates are seen, and they form a so-called “aortitis” pattern. **B**: At high magnification, plasma cell and eosinophil infiltrates are seen in the adventitia. **C-D**: In the retroperitoneum, plasma cell and eosinophil infiltrates are seen around **(C, D)** and within (**D**, arrowheads) the peri-aortic nerve ganglions.

The retroperitoneal mass was thickly fibrous, approximately 2 cm in diameter, and located around the left internal iliac artery. This mass was broadly fibrotic with infiltrations of plasma cells and eosinophils, which were also consistent with IgG4-RD. The inflammatory cells were distributed around the peripheral nerves and infiltrated the nerve fascicles (Figure 4). The abdominal aorta was also infiltrated with plasma cells and eosinophils, mostly in the adventitia and partially within the media, forming a “periaortitis” pattern [8]. Near the abdominal aorta, small foci of retroperitoneal fibrosis were observed, with infiltrations of plasma cells around and into the abdominal nerve plexus and ganglions (Figure 5).

**Figure 6:**
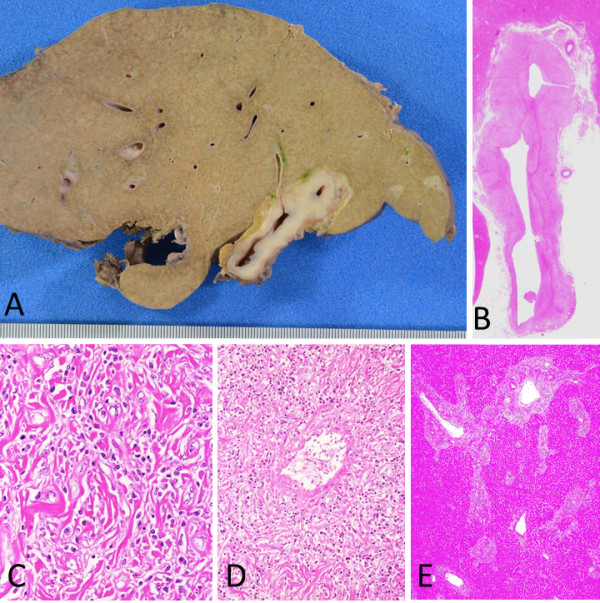
**Liver and extrahepatic bile duct. A**: Massive fibrosis occupies the hepatic portal area and extends until the extrahepatic bile duct and into the peripheral liver. **B**: Massive fibrosis extends from the hepatic portal area to the extrahepatic bile duct. **C**: In the hepatic portal site, plasma cell and eosinophil infiltrates are seen with storiform fibrosis. **D**: Obliterative phlebitis is seen throughout the liver. **E**: Fibrosis and plasma cell infiltration are seen in the portal area of the peripheral liver.

The liver weighed 980 g. Glisson’s sheath, at the hepatic portal site, was massively occupied with storiform fibrosis, fibroblasts, and infiltrations of IgG4^+^ plasma cells and eosinophils around the large bile ducts and vessels. This hepatic portal lesion extended to the portal areas of peripheral liver; the bile ducts were generally spared and bile stasis was rarely observed. However, obliterative phlebitis was frequently observed in the peripheral portal veins. The hepatic portal site lesion continued to the upper and middle regions of the extrahepatic common bile duct, which was also thickened with fibrosis and inflammatory cells, forming a secondary sclerosing cholangitis (Figure 6). There was no evidence of IgG4-RD in the common bile duct and pancreas.

**Figure 7:**
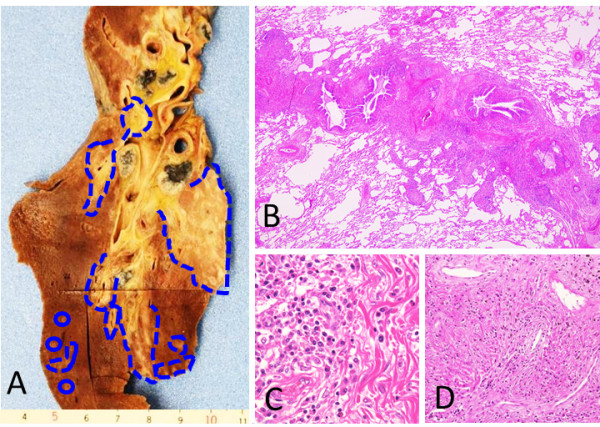
**Right lung. A**: Macroscopically, the peri-bronchial areas are densely fibrotic (blue dashed lines). **B**: Inflammatory cells and fibrosis are distributed along the bronchovascular bundles. **C**: Plasma cell and eosinophil infiltrates. **D**: An obstructed vessel is located in the pulmonary hilar region.

The left lung weighed 395 g and the right lung weighed 1018 g. Both lungs were the targets of massive IgG4-RD lesions. In each lung, infiltrations of plasma cells and eosinophils were observed, in conjunction with fibrosis, around the bronchovascular bundles. Some of the vessels were also observed to be obstructed by fibrosis (Figure 7). Plural effusion and edema were primarily located in the right lung, although serous, bilateral pleural effusions (150 mL) were also seen.

**Figure 8:**
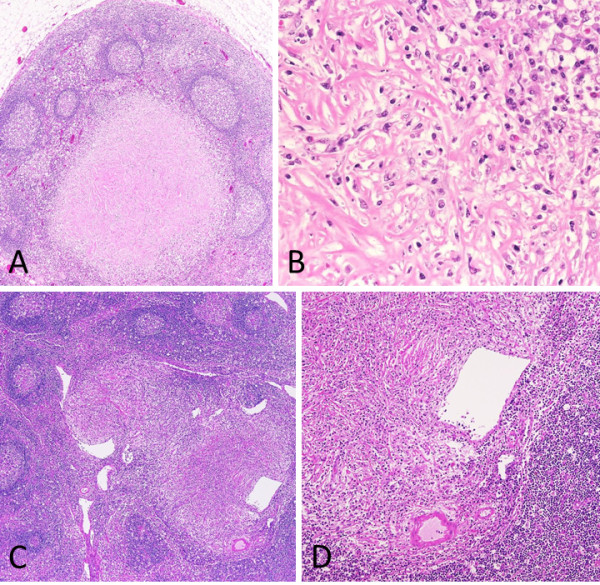
**Lymph nodes (A-B: peri-tracheal, C-D: peri-choledochal). A**: An IPT-like fibrotic nodule is seen. **B**: At high magnification, plasma cell and eosinophil infiltrates are seen around the IPT-like nodule with storiform pattern fibrosis. **C-D**: Obstructive phlebitis is seen in a peri-choledochal lymph node.

The lymph nodes in the mediastinal, bilateral pulmonary hilar, para-aortic, peri-choledochal, and peri-pancreatic regions were enlarged. The mediastinal and peri-choledochal lymph nodes contained massive sclerotic fibrosis with infiltrations of plasma cells, showing an IPT-like feature. Obliterative phlebitis was also seen within the peri-choledochal lymph nodes (Figure 8).

**Figure 9:**
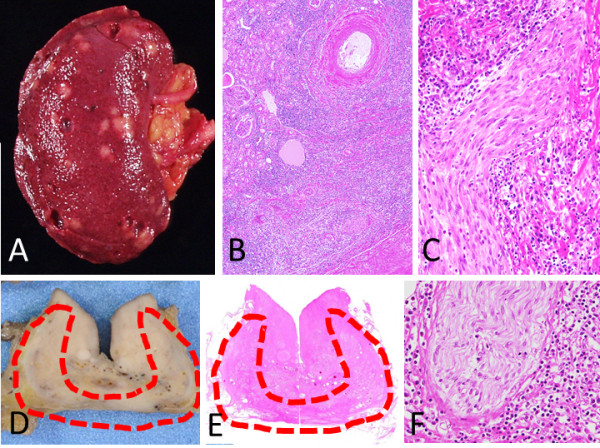
**Right kidney (A-C) and prostate (D-F). A**: Multiple white patchy lesions are seen in the renal cortex. **B**: A focal IgG4-RD lesion is seen in the renal cortex. **C**: Inflammatory cell infiltrates around the peripheral nerves in the renal pelvis. **D-F**: In prostate, although macroscopically obscure **(D)**, inflammatory cells infiltrate the peripheral zone (**D** and **E**, red dashed lines), whereas plasma cells and eosinophils infiltrate around and into the peripheral nerves **(F)**.

**Figure 10:**
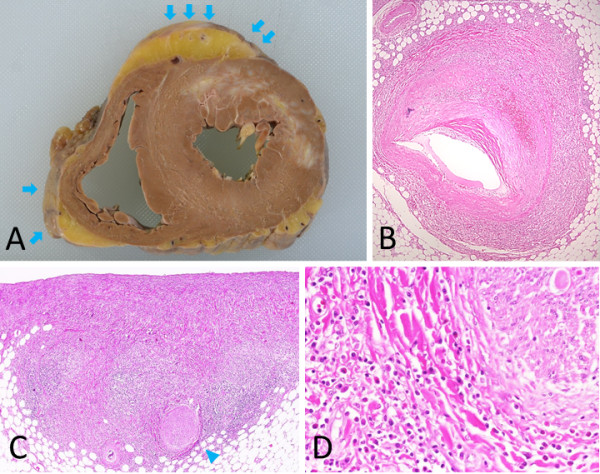
**Heart (A), coronary artery (B) and epicardium (C-D). A**: In horizontal section of the heart, an old myocardial infarction lesion (10 × 4 cm) is present from the anterior to the lateral walls. The epicardium is patchily fibrotic (arrows). **B**: At a strictured site of the circumflex branch of the left coronary artery, both atherosclerosis and massive plasma cell infiltration are seen, but they are not associated with each other. **C-D**: Epicardium. Plasma cells infiltrate around the peripheral nerves (arrowhead).

IgG4-related lesions were observed in the bilateral kidneys, urinary bladder, periphery of the prostate (Figure 9), epicardium, bilateral coronary arteries (Figure 10), and skin. In each location, the lesions were characterized by the presence of extensive infiltrations of plasma cells and eosinophils, especially around and into the peripheral nerves.

Marked, systemic atherosclerosis was present in the abdominal aorta and bilateral coronary arteries, and marked arterio-arteriolar nephrosclerosis was also seen. The heart weighed 340 g, and a 10 × 4 cm lesion from the old myocardial infarction was noted in the anterior to lateral walls of the left ventricle. Metallic stents were evident in the anterior descending and circumflex branches of the left and right coronary arteries, and the proximal parts of the stented sites were partially strictured by intimal atheromas and calcifications. Inflammatory cells, characteristic of IgG4-RD, were seen around the peripheral nerves in the epicardium; epicarditis was also evident (Figure 10).

No significant lesions were observed in the spinal cord or involving the intraspinal peripheral nerves.

## Discussion

In this patient, the submandibular lymph node that was excised 3 years previously was eventually proven to be PTGC-type IgG4-related lymphadenopathy, whereas some of the mediastinal and peri-choledochal lymph nodes showed an IPT-like feature with obliterative phlebitis [1-4,9]. Thus, 2 different types of IgG4-related lymphadenopathy were confirmed in 1 person over time. These observations suggest that PTGC-type IgG4-related lymphadenopathy, initially asymptomatic and localized, progressed to systemic lymphadenopathy and extranodal lesions over a 3-year period. The obliterative phlebitis that was seen in the peri-choledochal lymph nodes was also noteworthy, as obliterative phlebitis is rarely observed in lymph nodes [5].

As part of the differential diagnosis of IgG4-related lymphadenopathy, malignant lymphomas or lymphoproliferative disorders need to be excluded [10,11]. In this case, these diseases were ruled out because no monoclonality was detected.

In this patient’s extranodal lesions, the characteristic distribution of plasma cells and eosinophils was preferentially around the peripheral nerves, indicating that these lesions are a type of IgG4-related perineural disease, similar to the IgG4-RD cases reported with orbital and paravertebral localization [6,7]. Although the mechanism by which IgG4-RD involves multiple organs is still unclear, perineural extension may be associated with its spread throughout the body. The infiltration of plasma cells and eosinophils within nerve fascicles was an additional feature of this perineural disease. This patient also presented with other neurological symptoms, including constipation, chronic dysuria, and orthostatic hypotension. The presence of IgG4-RD lesions within the nerve fascicles and plexuses may help to explain the observed symptoms. Lesions involving the para-aortic nerve plexus and the destruction of the intramuscular nerve plexus of the duodenum and sigmoid colon can clearly cause peristaltic malfunctions, leading to constipation. Additionally, the prostate lesions may explain the chronic dysuria.

Nonocclusive mesenteric ischemia (NOMI), or “intestinal gangrene in the presence of a patent arterial tree”, is an uncommon condition in which severe, unrelenting, microvascular vasoconstriction results in bowel ischemia in the presence of pulsatile macroscopic arterial blood flow. NOMI is manifested by abdominal pain, usually occurring in patients with known atherosclerotic heart diseases [12,13]. In the present patient, many of the IgG4-RD abdominal lesions may have been associated with NOMI. The IgG4-related perineural disease and the destruction of Auerbach’s plexus of the sigmoid colon and duodenum may have caused the peristaltic malfunctions that led to the observed constipation. Obliterative phlebitis and the organization of the accompanying arteries in the sigmoidal mesentery likely resulted from the long-standing IgG4-RD mesenteric inflammation. These IgG4-RD lesions, in conjunction with plexus destruction, constipation, vessel lesions, and a post-myocardial infarction state, may have intertwined to produce the vasoconstriction that led to NOMI and, subsequently, to the patient’s death. In considering which vasoconstriction site led to NOMI, the superior and inferior mesenteric arteries were likely involved. The necrosis was prominent in the left-sided colon and was most severe in the sigmoid colon, indicating that the inferior mesenteric artery was the most crucial vasoconstriction site.

In the liver, although IgG4-RD caused secondary sclerosing cholangitis, bile stasis was minimal. Therefore, obliterative phlebitis of the portal veins, rather than cholangitis, may explain the elevated liver enzyme levels. In contrast to the hepatic or the upper and middle common bile ductular lesions, the lower common bile duct and pancreas were spared, which is not typical in cases of IgG4-RD.

In summary, different morphological types of IgG4-related lymphadenopathy were seen in a single patient. These lesions included a PTGC-type feature that developed 3 years prior to the determination of an IPT-like feature, suggesting that one type of IgG4-related lymphadenopathy may progress into another morphological type. Obliterative phlebitis was also noticed within the patient’s lymph nodes at autopsy. Another important feature was the presence of IgG4-related lesions around the peripheral nerves in several organs, which may be considered as IgG4-related perineural disease. The intestinal lesions of this patient may have been responsible for his end-stage constipation and ischemia. Moreover, the infiltration of plasma cells through the epineurium into the nerve fascicles has not been previously reported in the English literature. The reasons why inflammtory cells preferentially distribute around and into the peripheral nerves, and how often and to what extent IgG4-RD can cause neurological symptoms, remain to be elucidated.

## Conclusion

We encountered an autopsy case of systemic IgG4-RD with extensive involvement of the peripheral nerves. This case indicated that IgG4-RD may affect systemic organs through extensive peripheral nerve involvement.

## Consent

Written informed consent was obtained from the patient's family for publication of this Case Report and any accompanying images.

## Abbreviations

IgG: Immunoglobulin G; IgG4: Immunoglobulin G4; PTGC: Progressively transformed germinal centers; NOMI: Non-occlusive mesenteric ischemia; IPT: Inflammatory pseudotumor.

## Competing interests

The authors declare that they have no competing interests.

## Authors’ contributions

YS conceived and designed the study. YS, MF, NO, and TY made the pathological diagnosis and wrote the paper. KH, HK, and YK collected clinical information. All authors read and approved the final manuscript.
